# Local Anæsthesia

**Published:** 1867-09

**Authors:** D. Scott

**Affiliations:** Bellefontaine, Iowa


### LOCAL ANÆSTHESIA.

BELLEFONTAINE, Iowa, Aug. 4th, 1867.

N. S. DAVIS, M.D.:

*Dear Sir:*—My attention was called to the new mode of producing local anæsthesia, (in the number of the MEDICAL EXAM-

INER, for August, 1866,) invented by Dr. RICHARDSON, of London, and was determined to give it a trial. Accordingly, I purchased an ayparatus, and commenced experiments. I first tried it for destroying sensation in the teeth, for the purpose of extracting without pain. The result was very unsatisfactory in every case, owing to the abundance of saliva in the vicinity of the molars. It was almost impossible to accomplish freezing, and when I succeeded in doing so it was followed by no diminution in the pain of extraction, besides the operation itself being very painful. I next tried it for the purpose of making incisions of a necrosed portion of the ostibia, in a young man, but without success; the narcotism never penetrating to the depth of over $\frac{1}{16}$ of an inch. I then tried it in a case of carbuncle; then a common boil, and lastly in a case of whitlow, after which I abandoned it in disgust. The fluid used was rhigoline. I have very frequently tried it on my own fingers, and found it very painful, besides being followed every time by inflammation to some extent. Such is my brief experience with the spray producer, from which I pronounce the whole thing a failure.

Respectfully yours, D. SCOTT, M.D.

